# Herpes simplex virus encephalitis in pregnancy - a case report and review of reported patients in the literature

**DOI:** 10.1186/s13104-015-1071-6

**Published:** 2015-04-01

**Authors:** Katherine C Dodd, Benedict D Michael, Besa Ziso, Bode Williams, Ray Borrow, Anita Krishnan, Tom Solomon

**Affiliations:** Royal Preston Hospital, Sharoe Green Lane North, Preston, PR2 9HT UK; The Walton Centre Neurology NHS Foundation Trust, Lower Lane, Fazakerly, Liverpool, L9 7JL UK; Institute of Infection and Global Health, University of Liverpool, The Ronald Ross Building, West Derby Street, Liverpool, L69 7BE UK; Liverpool Women’s Hospital, Crown Street, Liverpool, L8 7SS UK; Vaccine Evaluation Unit, Public Health England, Oxford Road, Manchester, M13 9WZ UK

**Keywords:** Encephalitis, Herpes simplex, Encephalitis, Viral, Pregnancy complications, Infectious, Immunology

## Abstract

**Background:**

Herpes simplex virus (HSV) encephalitis is the most common sporadic cause of encephalitis with significant morbidity and mortality that is drastically reduced by early antiviral treatment.

**Case presentation:**

We report a 37 year old woman, 33 weeks pregnant, who presented with seizures due to proven HSV-1 encephalitis, and who had had a previous episode of probable viral encephalitis aged 14 years. She was successfully treated with aciclovir on both occasions and, in the latter, went on to deliver a healthy infant. This case is compared with 17 cases of HSV encephalitis in pregnancy in the literature identifying a predominance in the late 2nd and 3rd trimesters, perhaps in part due to immunological changes in pregnancy. The clinical presentation is also compared with non-pregnant patients with HSV encephalitis in the largest prospective UK and European studies. We also present practical advice on management from recent national guidelines.

**Conclusion:**

When pregnant women present with new seizures, headache, impaired consciousness or altered behaviour urgent investigation is required to identify common diagnoses, such as eclampsia, venous sinus thrombosis and metabolic disturbances. Nevertheless, viral encephalitis is a very treatable cause of this presentation with potentially serious complications if missed, and may be more common in latter stages of pregnancy. Encephalitis should not be discounted if the patient is afebrile, has a normal Glasgow coma score, or the cerebrospinal fluid white cell count is only slightly elevated, as these features are well recognised in viral encephalitis.

## Background

Herpes simplex virus is the most common sporadic cause of encephalitis, with the majority of cases due to HSV type-1. Early investigation and treatment is critical, as mortality is reduced from >70% to <20-30% [1,2]. HSV encephalitis has been described in pregnancy but little is known about how the clinical presentation and outcome compare between this group and those with HSV encephalitis outside of pregnancy.

## Methods

To assess if the clinical presentation and outcome in pregnant patients differs from non-pregnant patients, we first describe a case of HSV encephalitis in pregnancy. In addition, a Medline search for previous case reports of HSV encephalitis in pregnant women in English (between 1972 and 2013) was performed, using the search terms herpes simplex encephalitis and pregnancy. Cases with HSV type 1 and type 2 were identified, but were only included if they met established clinical case definitions for encephalitis, namely encephalopathy (altered consciousness that persisted for longer than 24 hours, including lethargy, irritability, or a change in personality and behaviour) and two or more of the following: fever or history of fever (≥38°C) during the presenting illness; seizures and/or focal neurological findings (with evidence of brain parenchyma involvement); CSF pleocytosis (>4cells/μL); electroencephalographic findings indicative of encephalitis; and abnormal results of neuroimaging (computed tomography or magnetic resonance imaging) suggestive of encephalitis [3,4]. Data from these cases were compared with those from the two largest multicentre prospective studies of HSV encephalitis in the UK and Europe; the authors of the UK paper were approached to provide additional raw data when required [4,5]. Univariate analysis was performed using the chi-squared Fisher’s exact test for categorical variables and the Mann–Whitney U test for continuous data using SPSS 2012^©^. A Glasgow coma score (GCS) of <8 was defined as coma and statistical significance as a p value of <0.05.

## Case presentation

A 37 year old right-handed woman, who was 33 weeks pregnant (gravida 5, para 3), presented following a first seizure. She had a preceding 10-day history of diarrhoea, vomiting, photophobia and severe headache, which woke her up from sleep and was exacerbated by coughing. There was no recent travel or head injury. A witness reported that she had visual hallucinations, followed by an episode of inappropriate laughter and within minutes a secondary generalised seizure: her right arm moved over her chest and her head deviated to the right, followed by her whole body becoming stiff, with clonic movements of all limbs. She became cyanosed, bit her tongue, had urinary incontinence and was unrousable. This lasted for approximately five minutes, followed by post-ictal drowsiness for two hours.

At presentation to the local teaching hospital she was apyrexial, had a GCS of 15/15, and normal cardio-respiratory and neurological examinations. She was not hypertensive and urinalysis did not demonstrate proteinuria. Her full blood count, renal, liver and bone profiles were normal, the erythrocyte sedimentation rate was 39 (reference range 5–15) mm/hr and C-reactive protein was 6 (<10) mg/L. Magnetic resonance imaging (MRI) and venography of the brain on day one were normal. Oral carbamazepine was commenced and, following a second tonic-clonic seizure 16 hours after admission, oral clobazam was added.

A lumbar puncture (LP) demonstrated an opening pressure of 14 (reference range 7–18) cm, CSF white cells count (WCC) 10 (<5) ×10^6^/L, with predominance of immature neutrophils (percentage not known), red cell count (RCC) 2 (0–10) ×10^6^/L, protein 0.23 (0.15-0.4) g/L, and CSF glucose 3.6 (2.8-4.2) mmol/L, 67% of blood glucose. No organisms were seen, and oligoclonal bands were negative. On receipt of these results, intravenous aciclovir was commenced; approximately 25 hours after admission.

On day two she developed fluctuating confusion, abnormal behaviour and a temperature of 37.8°C. Her GCS deteriorated to 11/15 (eyes opening to speech, incomprehensible sounds and obeying commands), and she had a positive Babinski’s sign on the right. She was given intravenous phenytoin, for clinically suspected non-convulsive status epilepticus.

The patient had previously been admitted, when aged 14, with acute headache, confusion, vomiting, photophobia and diplopia. LP demonstrated a CSF protein of 0.8 (0.15-0.4) g/L, glucose 3.6 (2.8-4.2) mmol/L, WCC 1 (0–5) ×10^6^/mm^3^, RCC 38 (0–10) ×10^6^/L, and no organisms were seen. Viral polymerase chain reaction (PCR) analysis and antibody testing were not available. A computed tomography (CT) scan of the brain demonstrated small ventricles with early cerebral oedema. A full course of intravenous aciclovir was given for presumed HSV encephalitis. She made a full and uneventful recovery.

In the current admission, CSF PCR returned positive for HSV type-1 and negative for HSV type-2, varicella zoster virus and enterovirus. Electroencephalography (EEG) showed left fronto-temporal epileptiform discharges, and repeat MRI on day three showed classic changes of herpes simplex encephalitis with high signal on diffusion weighted imaging of the left temporal pole and left insular cortex.

The aciclovir was stopped after 16 days, following repeat CSF HSV-1 PCR testing negative. Foetal growth scans and foetal heart rate monitoring were normal throughout. An elective caesarean section at 39 weeks was undertaken with no complications, and a healthy boy, of 3.725 kg, was delivered. The neonate was not given aciclovir. A repeat EEG prior to discharge showed marked improvement albeit with some residual slowing. Phenytoin and clobazam were gradually tapered off and carbamazepine continued. At six month follow-up the patient is well with no seizure recurrence and the child was healthy.

## Results

21 cases were identified, four were excluded; two as they contained cases of metabolic encephalopathy and disseminated HSV-2 infection without encephalitis, and two had no definite proof of HSV infection [6-9]. Therefore, 17 cases were included in the analysis and these are summarised in Table 1 [7,10-23]. The majority of cases occurred in the later stages of pregnancy, with 11 (61) in the 3rd trimester, 6 (33%) in the 2nd trimester and only 1 (6%) in the 1st trimester.

**Table 1:** **Previous HSV encephalitis in pregnancy case reports**

**Year and Author**	**Age (yrs)**	**Gestation (wks)**	**Type**	**Admission**			**Investigations**			**Treatment**	**Outcome**
				**Febrile**	**GCS**	**Symptoms**	**CSF**	**CT/MRI**	**EEG**		
2015 Dodd KC (This case)	37	33	1	N	15	Headache, vomiting, photophobia, diarrhoea, visual hallucinations, confusion and seizures.	WCC 10	MRI - Initially normal, then increased signal in the left temporal lobe with cytotoxic oedema	Left fronto-temporal epileptiform changes	IV aciclovir 10 mg/kg TDS stopped after 16 days when repeat HSV PCR negative	Elective caesarean section at 39 weeks. Mother and child well at 5 months.
							HSV-1 PCR				
2012. Pascal J, et al. [10]	31	33	1	Y	13	Pyrexia, vomiting, headache, neck stiffness, photophobia, phonophobia, visual and auditory symptoms.	WCC 345	MRI - Hyperintensity with oedema in the right temporal area and cerebral peduncle	Not done	IV aciclovir 10 mg/kg TDS stopped after 21 days and repeat HSV PCR negative	Normal vaginal delivery at 39 weeks with epidural analgesia. Mother and child normal and healthy 15 months later.
							HSV-1 PCR				
Mesker AJ et al. 2013 Dodd KC, et al. [11]	30	37	1	Y	NK	Headache, fever, mental status change and reduced consciousness.	WCC unknown HSV-1 PCR	CT + MRI - Abnormalities in right temporal lobe	Not done	IV aciclovir 750 mg TDS (unknown course length)	Caesarean section at 37 weeks – healthy child.
										Dexamethasone 10 mg QDS 4 days	Patient improved but deficit in spatial orientation on discharge.
2009. Sellner et al.[12]	25	32	1	Y	NK	Tonic-clonic seizure, drowsy, headaches, photophobia, vomiting, antero- and retro-grade amnesia.	WCC 125	MRI - Right temporopolar and medial hyperintensity, with cytotoxic oedema	Not done	IV aciclovir 12.5 mg/kg TDS for 21 days. Stopped after repeat HSV PCR negative.	Caesarean section at 33 weeks due to deterioration.
							HSV-1 PCR				Mother and child healthy 4 weeks after discharge.
2008 Piskin N, et al.[13]	26	25	1	Y	NK	Fever, headache, nausea, mental status changes. Tonic-clonic seizure during admission.	WCC 70	MRI - Increased signal and oedema in the right temporal region.	Diffuse slowing with epileptic activity right frontal region.	IV aciclovir 750 mg TDS for 21 days	Normal vaginal delivery at term.
							HSV-1 PCR			Dexamethasone – reducing regimen 28 days	2 months later – MRI shows clear regression and repeat EEG normal.
2006 Gunduz A, et al. [14]	24	7	NK	Y (low grade)	NK	Headache, episodes of unresponsiveness, non-convulsive status epilepticus.	WCC Normal (figure not given)	MRI - Normal	Ictal state – nonconvulsive status	Aciclovir 30 mg/kg/day (unknown duration)	Patient improved and seizure free at 10 months on carbamazepine.
							HSV PCR				Pregnancy terminated.
2003 Godet C, et al.[15]	29	38 (post-partum)	2	Y	NK	Post caesarean section developed fever and then impaired consciousness and amnesia.	WCC 9	CT – normal	Normal	Intravenous aciclovir 10 mg/kg every 8 hours	The fever and neurological disorder resolved after a few days on aciclovir.
							HSV-2 PCR				
1999 Dupuis O, et al.[16]	31	35	1	Y	NK	Headache, vomiting, and photophobia. Then confusion, aphasia, and auditory hallucinations.	WCC 138	CT - Normal	Epileptic foci in left temporal region	Intravenous aciclovir (unknown dose and duration)	Delivered a healthy child at term.
							HSV-1 PCR	MRI - Abnormal signal in the left temporal region			Three months later, the mother exhibited moderate amnesia.
1999 Dupuis O, et al.[16]	35	27	1	Y	15	Generalised seizure, fever, headache, and photophobia. Then confusion, followed by coma, right paraparesis and facial palsy.	WCC 156	CT - Normal	Abnormal signal in left frontotemporal region.	Aciclovir (unknown dose and duration)	Vaginal delivery at term. Child healthy.
							HSV-1 PCR	MRI - Increased signal in left temporal region.			Mother walking by day 23. Seizure recurrence at 12 weeks. At one year severe anterograde memory loss.
1992 Luby JP.[17]	15	35	1	Y	15	Fever, nausea, sore throat, and headache.	WCC 398	CT - A low density area in the right temporal lobe	Not done	Aciclovir 10 mg/kg TDS for 14 days.	Labour induced at 35 weeks. Discharged after 15 days – patient and infant healthy.
						Developed nystagmus, focal seizures and confusion.	Brain biopsy culture grew HSV-1				
1992 Anteby E, et al.[18]	28	21	NK	Y	NK	Fever and acute confusional state. Mild right hemiparesis.	WCC −0 initially,	CT – normal	Diffuse slowing, pronounced over the left parieto-temporal regions.	Aciclovir (2250 mg/day) for 10 days	Discharged in good health after 10 days. Delivery of normal child at 39 weeks.
							105 after 1 week.	MRI - normal			
							CSF serology – anti-HSV seroconversion 1:8 to 1:512				
1990 Frieden FJ, et al.[19]	37	26	1 + 2	Y	14	Headache, confusion, aphasia, right sided paraesthesias, and fever.	WCC 623 Antibody titres positive for type 1 (>1:1600) and type 2 (>1:400)	CT - Low density in the left temporal-parietal region	Diffuse bilateral cerebral dysfunction more prominent on the left	Aciclovir IV 500 mg TDS (unknown duration)	The patient improved gradually with treatment and discharged well on day 11. Forceps delivery at term of a healthy infant.
								MRI - Increased signal left temporal-parietal region		Methylprednisolone 25 mg IV QDS	
1989 Besser R et al.[20]	25	23	1	Y	15	Headache, vomiting and fever. Nuchal rigidity and somnolence developed.	WCC 320	CT - A large low-density lesion in the right temporal lobe sparing the lenticulate nucleus	Moderate slowing of background activity with delta waves in the right temporal region	Aciclovir 10 mg/kg TDS for 10 days	Premature labour required tocolysis.
							HSV-1 IgM and IgG (ELISA)				Improved rapidly and completely once aciclovir started. Delivered a healthy child 16 weeks later.
							HSV complement fixing antibodies in CSF rose from 1:2 to 1:16				
1987 Hankey GJ, et al.[21]	22	29	NK	Y	15	Fever, headache and malaise. Developed seizures and reduced GCS.	WCC 270	CT - Hypodense area in right temporal lobe with oedema	Diffusely abnormal, right side worse than left	Aciclovir 800 mg/day 22 days	Slow recovery over 2 months. Vaginal delivery at 41 weeks, healthy child.
							Serum HSV complement fixation antibody titres, and HSV-specific IgM in CSF.			Dexamethasone 4 mg QDS in a reducing regimen to 22 days	On-going secondary generalised seizures, but otherwise well.
1986 Berger SA, et al.[22]	41	32	2	Y	NK	Fever, confusion, seizures, and then stupor.	WCC 15 leucocytes	CT – diffuse cerebral oedema with bitemporal cerebral necrosis	Seizure foci in both temporal lobes	Aciclovir IV 10 mg/kg TDS on day 5 for 3 doses and then day 13 for 7 days.	Infant delivered day 18 by caesarean section. The child had disseminated HSV infection treated successfully with IV aciclovir. The mother died 2 days later.
										Adenine arabinoside IV 30 mg/kg 8 days.	
										Dexamethasone	
							ELISA of maternal and infant sera demonstrated antibody to HSV-2.				
1979 Roman-Campos G, et al.[7]	22	16	NK	N	NK	Spontaneous abortion, and 2 months of abnormal behaviour.	Post mortem – bitemporal necrotising encephalitis with intranuclear inclusion bodies in neurons. Electron microscopy - herpevirus particles.	Not done	Not done	None	Patient had curettage following admission, then developed shock and low GCS. Died on day three.
1979 Roman-Campos G, et al.[7]	17	24	NK	N	NK	Bizarre behaviour, a week later coma and then seizures.	WCC 0	Carotid angiogram negative	Generalised low voltage slow activity in the temporal regions.	Dexamethasone	Delivered a macerated foetus after one week. Patient died after 2 weeks.
							Brain biopsy – brain oedema and necrotising encephalitis with multiple internuclear inclusion bodies. Electron microscopy - herpesvirus particles.				
1972 Anderson JM, et al.[23]	19	~39	NK	Y	15	Pharingitis and fever, followed by dysphasia, right hemiparesis, paraesthesia, hemianopia and reduced conscious level.	CSF WCC not stated.	Technetium brain scan – a large left temporal space occupying lesion.	Not stated	Idoxuridine 2.5 g TDS started after 10 days	The patient died after twelve days. She delivered a healthy live child five days before she died.
							Culture – HSV.				
							Brain biopsy –positive fluorescent antibody study to HSV				

Abbreviations: *CSF*: Cerebrospinal fluid, *IV*: Intravenous, *MRI*: Magnetic resonance imaging, *NK*: Not known, *WCC*: White cell count.

In this case, the patient was afebrile and the CSF white cell count was only slightly raised. In comparison to the largest prospective UK cohort of 38 patients and the European cohort of 98 patients, the proportion of patients during pregnancy reported in the literature who were afebrile on admission was similar (Table 2). The mean WCC was also similar between the groups. In this case, although there had been a preceding seizure, at the time of initial clinical clerking assessment the GCS was normal. Of the cases in the literature at presentation, none of the eight pregnant patients in whom the GCS was available had GCS <8, whereas this has previously been seen in 24% of the general mixed sex population with HSV-1 encephalitis and 9% of adults with HSV encephalitis overall [4,5]. This may reflect an earlier presentation in pregnant patients, as the median (range) days of symptoms prior to admission was shorter for pregnant patients, although this was not statistically significant (3 [0–10] vs 6.5 [0–30], p = 0.35). There are limitations in the interpretation of this data as the case reports are from a wide time period and there is variability in the data that is presented within them. What would have been ideal, but was not possible due to the limitations of the published data, would have been to compare the time from onset of symptoms, to onset of coma between pregnant patients with HSV1 encephalitis and an age and sex-matched cohort with HSV1 encephalitis.

**Table 2:** **Comparison between clinical features, investigation findings and outcome of HSV encephalitis in the general population and those pregnant**

	**Pregnant patients with HSV encephalitis**	**General population with HSV-1 encephalitis [** 4 **]**	**p value**	**General population with HSV encephalitis [** 5 **]**	**p value**
Fever	89% (16/18)	76% (29/38)	0.75	92% (90/98)	0.22
Headache	67% (12/18)	42% (16/38)	0.1	NK	NK
Seizures	45% (8/18)	63% (24/38)	0.17	32% (31/98)	0.32
Mean GCS on admission (where documented)	14.6	NK	NK	13.7	NK
Coma on admission (GCS < 8, where GCS documented)	0% (0/8)	24% (9/38)	0.32	9% (9/98)	0.36
Abnormal CT	40% (4/10)	56% (18/32)	1.0	79% (72/91)	0.17
(if performed)					
Abnormal MRI	80% (8/10)	89% (25/28)	0.59	NK	NK
(if performed)					
Abnormal EEG	92% (11/12)	81% (22/27)	0.64	NK	NK
(if performed)					
WCC mean (range)	177 (0–623)	46 (10–1278)	0.51	237 (1–3900)	NK
Death	3/18 (17%)	11% (4/38)	0.67	13/85 (15%)	1.0

Footnote: Population 4: Granerod et al [4]; Population 5: Raschilas et al [5].

The outcome of pregnant patients treated for HSV encephalitis appears not to be statistically different to that in the general population. In those patients treated with aciclovir (n = 15), only 1 women (7%), and 1 foetus, died with on-going disability for 4 (27%) of the surviving women. Nevertheless, of those not given aciclovir (n = 6), 4 (67%) women and 3 (50%) foetuses died. In other studies, mortality in HSV encephalitis treated with aciclovir has been reported as 11% – 15%, without a significant difference in outcome between sexes, although this may reflect publication bias [4,5].

## Discussion

Herpes simplex virus is the most common cause of sporadic viral encephalitis with 90% of cases being due to type-1 [1,4]. It usually presents as a concurrent or antecedent febrile or coryzal illness with headache, impaired cognition, reduced consciousness, changes in personality and behaviour and seizures.

This patient had proven HSV-1 encephalitis when pregnant aged 37, with an episode of encephalitis aged 14 which was of uncertain aetiology due to investigatory limitations at the time of intitial presentation in childhood, although HSV would be the most common cause and the patient was treated for presumed HSV encephalitis. Therefore, this may possibly represent a late relapse of HSV-1 encephalitis during pregnancy, which has not previously been reported, although more thorough investigation at the time of the childhood illness would be required to establish this. Relapse of HSV-1 encephalitis is rare, but recognised, and can be either early, due to suboptimal treatment or post-infectious immune process, or late, with latencies previously reported of up to 8.5 years [2,24].

During pregnancy, women may be at an increased risk of certain infections, and also increased risk of severe manifestations of infection [25-27]. There are many complex immunological adaptations during in pregnancy, to prevent rejection of the ‘foetal allograft’, which contains paternal antigens [25,26]. Oestrogens and progesterones rise during pregnancy, are highest in the late 2nd and 3rd trimesters, and are thought to modulate an immunological shift, in both the cell-mediated and humoural systems [25,26]. Interestingly the vast majority of cases of HSV encephalitis identified in pregnancy occurred in the 3rd trimester and, to a lesser extent in the 2nd trimester, with only 2 occurring in the 1st trimester. This may, however, relate to lower recognition of pregnancy during the first trimester.

**Figure 1:**
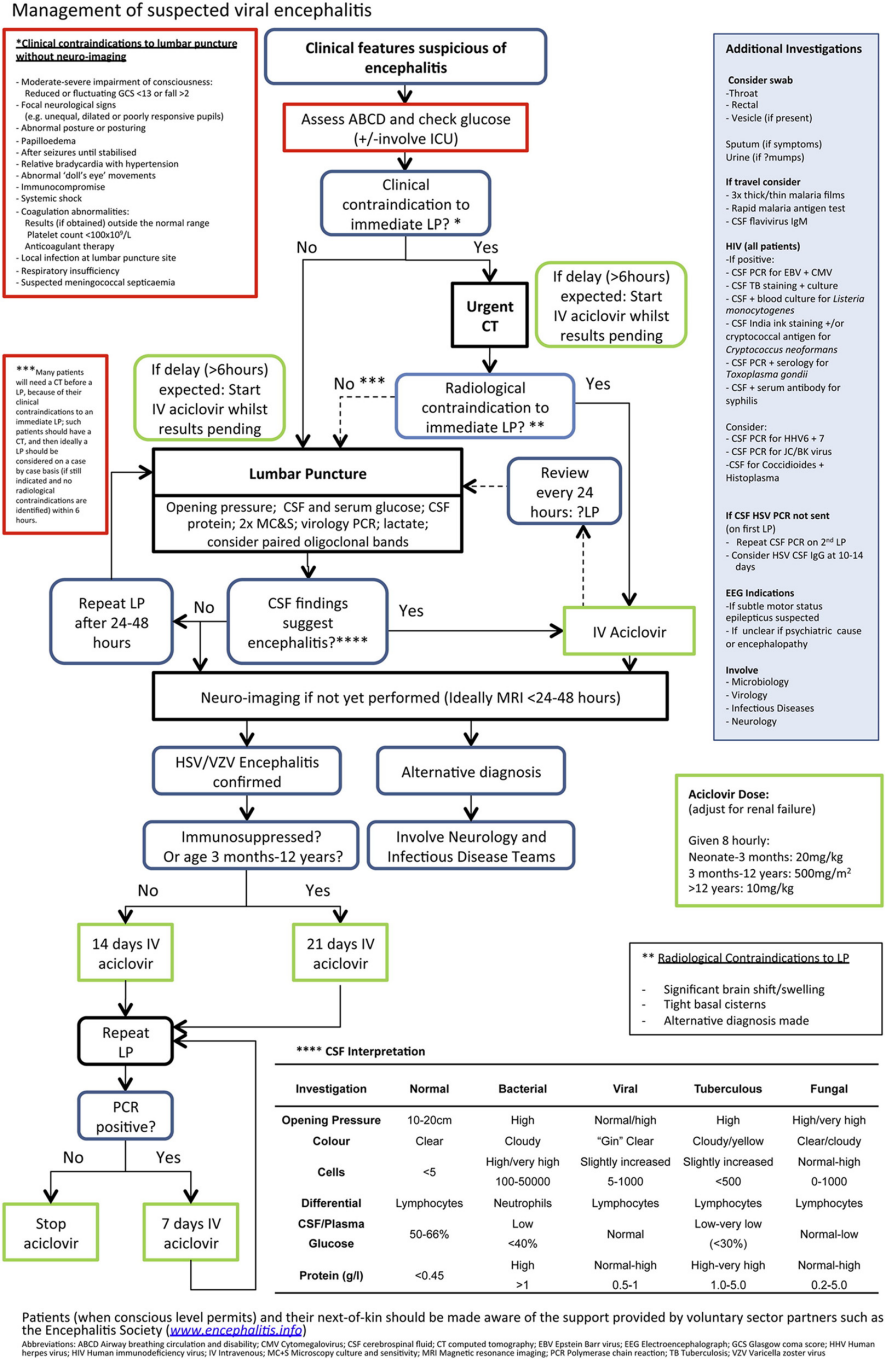
**Algorithm for the management of patients with suspected viral encephalitis.** (Reprinted from Journal of Infection, 64(4): 347–73, Solomon T, Michael BD, Smith PE, et al. Management of suspected viral encephalitis in adults - Association of British Neurologists and British Infection Association National Guidelines, Copyright 2012, with permission from Elsevier) [1].

**Figure 2:**
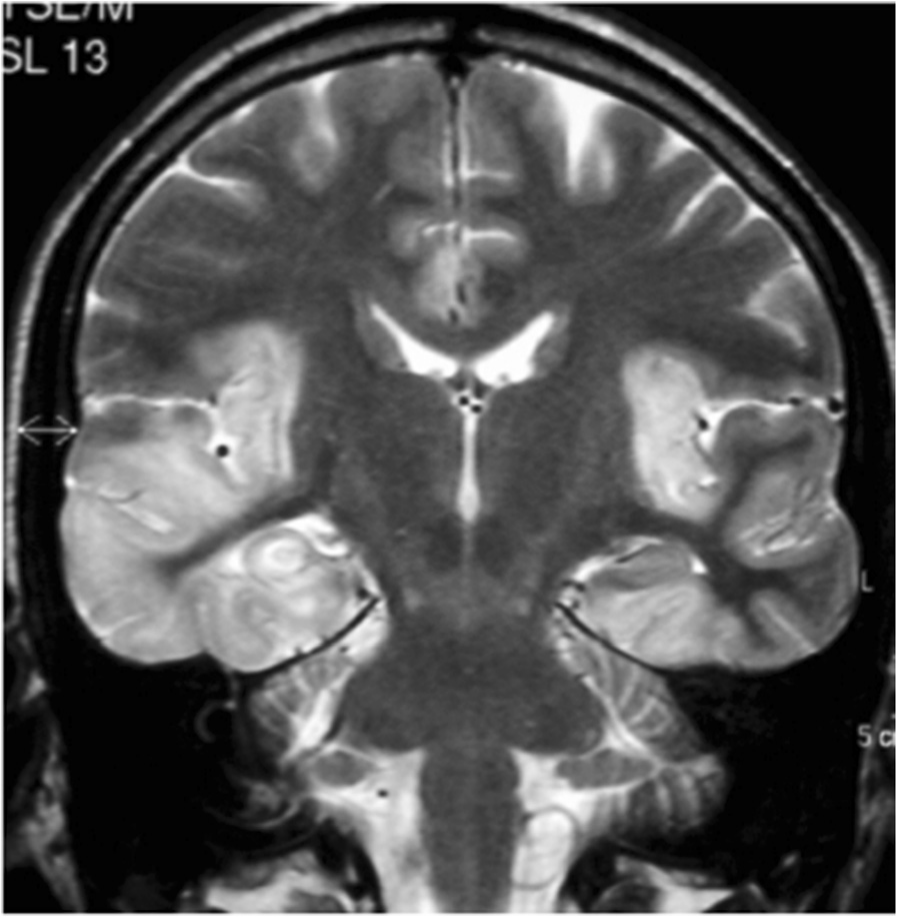
**Classical asymmetrical temporal lobe T2 hyperintensity in a patient with Herpes Simplex Virus type-1 encephalitis.**

In pregnant women presenting with seizures, headache, or altered behaviour, in addition to viral encephalitis the differential diagnosis includes and number of other structural and metabolic causes which must be excluded. Hyperemesis gravidarum in early pregnancy results in excessive vomiting producing electrolyte imbalance, which, if severe, may provoke seizures [28]. Eclampsia refers to seizures or coma as severe complications of pre-eclapmsia; this is identified by hypertension and proteinuria. Pregnancy increases blood viscosity and the risk of thrombosis; therefore if seizures were preceded by headache, especially if with features of raised intracranial pressure, then an MR venogram should be performed. Further differential diagnosis includes acute hepatitis, malaria, ischaemic stroke, or acute intermittent porphyria [29]. The UK guideline for the management of suspected viral encephalitis recommend intravenous aciclovir as soon as possible if the CSF or MRI findings suggest viral encephalitis, or within 6 hours if these results are not available (Figures 1 and 2) [1,27]. There are no specific guidelines for management of viral encephalitis in pregnancy, but there is accumulating evidence that aciclovir is safe in pregnancy and is not associated with an increase in birth defects [30]. As mortality in HSV encephalitis is reduced from >70% to <20-30% with aciclovir, and delay in starting treatment is associated with a worse outcome, treatment should be started promptly in all patients with suspected HSV encephalitis [1,27,31]. In pregnant patients with seizures the lowest effective dose of anti-epileptic drugs is preferable, avoiding polytherapy and particularly potentially teratogenic drugs [32]. Nevertheless, the aim should be seizure freedom as there is a risk to the foetus during tonic-clonic seizures [32]. There is currently no evidence to support the use of anti-epileptic drugs as primary prophylaxis in viral encephalitis, and no evidence to direct secondary prophylaxis [33,34].

We identified that the cases of HSV encephalitis reported in pregnancy often did not have a significantly impaired GCS and that this may reflect an earlier presentation of some patients in this group. However, there are limitations in the interpretation of these data as the case reports are from a wide time period and there is variability in the data that is presented within them. What would have been ideal, but was not possible due to the limitations of the published data, would have been to compare the time from onset of symptoms, to onset of coma between pregnant patients with HSV1 encephalitis and an age and sex-matched cohort with HSV1 encephalitis.

## Conclusion

Urgent investigation for common diagnoses, such as eclampsia, venous sinus thrombosis and metabolic disturbance is required when pregnant women present with headaches, new seizures, and changes in cognition or behaviour. HSV encephalitis is an additional treatable cause of this presentation, with potentially devastating consequences if missed, and may be more common in the late 2nd and 3rd trimesters. Importantly, HSV encephalitis should not be discounted in pregnant patients presenting with a normal or near-normal GCS, which can be seen in all patients with HSE, but may be more common during pregnancy; possibly due to the altered immune response, or earlier presentation. In future, where possible, pregnant women should not be excluded from studies of immunological responses during viral encephalitis.

## Consent

We have obtained the patient’s full consent for publication. Consent for anonymous publication of the index case in this report was obtained by the authors, consent for the additional cases in this series is provided with the individual case publications.

## Abbreviations

HSV: Herpes Simplex Virus
GCS: Glasgow Coma Score
CSF: Cerebrospinal Fluid
MRI: Magnetic Resonance Imaging
LP: Lumbar Puncture
WCC: White cell count
RCC: Red cell count
PCR: Polymerase chain reaction
CT: Computed tomography
EEG: Electroencephalogram
